# Non-linear associations of HOMA2-IR with all-cause mortality in general populations: insights from NHANES 1999–2006

**DOI:** 10.1186/s12889-024-18026-7

**Published:** 2024-02-22

**Authors:** Aikai Zhang, Lingchen Huang, Min Tang

**Affiliations:** 1 Department of Cardiology, State Key Laboratory of Cardiovascular Disease, Cardiovascular Institute, Fuwai Hospital, National Center for Cardiovascular Diseases, Chinese Academy of Medical Sciences, Peking Union Medical College, 100037 Beijing, China; 2 Department of Cardiac Surgery, State Key Laboratory of Cardiovascular Disease, Cardiovascular Institute, Fuwai Hospital, National Center for Cardiovascular Diseases, Chinese Academy of Medical Sciences, Peking Union Medical College, 100037 Beijing, China

**Keywords:** Homeostatic model assessment, Insulin resistance, Obesity, NHANES

## Abstract

**Background:**

The association between homeostatic model assessment (HOMA2-IR) and mortality in obese and non-obese populations has not been clearly explained.

**Methods:**

A total of 7,085 individuals aged ≥ 20 years from the 1999–2006 National Health and Nutrition Examination Survey were included in the study. Study endpoints were all-cause and cardiovascular mortality. Multivariate Cox proportional hazards regression models with restricted cubic spline analysis were used for analysis.

**Results:**

In the study populations, a total of 1666 all-cause deaths and 555 cardiovascular (CV) deaths were recorded during a mean follow-up of 195.53 months. Notably, a significant difference in obesity was observed in the association between HOMA2-IR and mortality. After adjustment for multiple variables, HOMA2-IR was positively associated with all-cause mortality in all participants, in those with normal BMI, and in those with obesity. Conversely, tertile 2 of HOMA2-IR was associated with a lower risk of all-cause mortality in participants with obesity compared with tertile 1 (adjusted hazard ratio, 0.68; 95% confidence interval, 0.52–0.89; *P* = 0.005). Results from restricted cubic spline analysis showed a J-shaped association between HOMA2-IR and all-cause and CV mortality. In addition, a nonlinear U-shaped correlation with all-cause (*P* for nonlinear < 0.001) and CV (*P* for nonlinear = 0.002) mortality was observed in the population with obesity, with inflection points of HOMA2-IR identified at 1.85 and 1.75. Below the inflection point of 1.85, a negative relationship between HOMA2-IR and all-cause mortality was observed.

**Conclusions:**

Elevated HOMA2-IR showed a notable correlation with increased risk of all-cause mortality. It was noteworthy that excessively reduced levels of insulin resistance showed a distinct association with increased mortality in individuals with obesity.

**Supplementary Information:**

The online version contains supplementary material available at 10.1186/s12889-024-18026-7.

## Introduction

Insulin resistance (IR) is emerging as an important factor associated with increased susceptibility to cardiovascular (CV) disease and type 2 diabetes mellitus (T2DM). In addition, IR is a central component within the diagnostic framework of metabolic syndrome (MS) [1]. The prevalence of MS has been steadily increasing, in line with the rising prevalence of obesity worldwide [2].

The association of homeostatic model assessment (HOMA2-IR) was updated in 1998 and has been proved to perform better than the original HOMA in assessing IR or β-cell function and predicting T2DM progression [3, 4]. This refined model has been carefully recalibrated to account for shifts in plasma glucose-insulin dynamics, particularly in cases where fasting plasma glucose (FPG) exceeds the 10 mmol/L threshold. The distinguishing feature of HOMA2-IR is that it illuminates the intricate, non-linear interplay between plasma glucose, insulin, and IR, providing a more comprehensive understanding of these complex relationships [5]. The discriminatory threshold of HOMA2-IR for the identification of IR remained fixed at 1.7. Notably, within the cohort of non-diabetic participants included in the Brazilian Metabolic Syndrome Study, an alternative threshold of 1.8 emerged as the recommended cut-off point for distinguishing IR by HOMA2-IR assessments [6, 7].

Obesity orchestrates a peculiar landscape characterized by elevated visceral adipose tissue, precipitating the release of an excessive cadre of free fatty acids, reactive oxygen species, and pro-inflammatory cytokines into extrinsic domains beyond adipose reserves. The resulting effects manifest as a disruption in the seamless choreography of insulin within intricate signaling pathways, thereby disrupting the delicate balance of glucose homeostasis and promoting the emergence of widespread systemic insulin resistance [8].

Previous studies have shown that both metabolically healthy obesity and metabolically abnormal obesity were associated with an increased risk of mortality compared with their healthy counterparts. Notably, within the obesity domain, the dynamics of all-cause and CV mortality remained relatively stable regardless of metabolic health [9]. In individuals with a body mass index (BMI) < 25 kg/m2, the risk of stroke and myocardial infarction was even higher in the highest quartile of IR than in the subgroup with a BMI ≥ 25 kg/m2 [10]. Reduced IR levels showed a striking correlation with reduced fasting glucose concentrations, a dynamic that could have potentially adverse implications. Taken together, these studies revealed a labyrinthine interrelationship among BMI, IR, and metabolic processes that exerts a multifaceted influence on mortality outcomes.

However, there is a lack of studies to elaborate on the long-term effects of IR, especially low IR level, in individuals contending with obesity. Here, data from the National Health and Nutrition Examination Survey (NHANES) were collected to investigate the effect of low HOMA2-IR levels on mortality in the individuals with obesity.

## Methods

### Study design and population

The data used in this study were all from the 1999–2006 NHANES database. NHANES is a periodic cross-sectional health survey program that uses a complex multistage probability sampling design to assess the health and nutrition status of adults and children in the United States. The Ethics Review Board of the National Center for Health Statistics approved the research protocol. Written informed consent was signed by all participants. This study followed the reporting guideline Strengthening the Reporting of Observational Studies in Epidemiology [11]. NHANES data used in this study can be extracted from DataDryad (10.5061/dryad.d5h62).

**Fig. 1:**
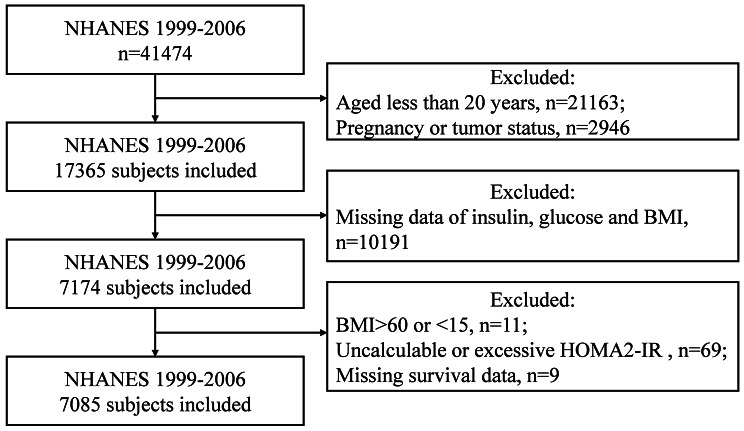
Flow chart of participants included from the NHANES 1999–2006

Of the 41,474 participants in NHANES between 1999 and 2006, we excluded 21,163 participants who were less than 20 years old. We also excluded participants with: (1) pregnancy status or tumor (*n* = 2946); (2) missing fasting serum insulin and glucose data (*n* = 10,040); (3) missing BMI data (*n* = 151); (4) extreme BMI (BMI < 15 or BMI > 60) (*n* = 11); (5) noncalculable HOMA2-IR (fasting serum glucose < 3. 0 mmol/L or > 25 mmol/L, fasting serum insulin < 20 pmol/L or > 400 pmol/L) or extreme HOMA2-IR (up to 1%) (*n* = 69); (5) missing survival data (*n* = 9). The final study included 7085 adult subjects (Fig. 1).

### Exposure variable and outcome variables

The exposure variable was the HOMA2-IR, which was mathematically derived from the calculator obtained from the website(https://www.dtu.ox.ac.uk/homacalculator/). Based on the value of HOMA2-IR, all individuals were divided into three tertiles: tertile 1(HOMA2-IR < 0.82), tertile 2(0.82 ≤ HOMA2-IR < 1.45), tertile 3(HOMA2-IR ≥ 1.45). Participants with BMI < 25 kg/m2 were defined as having normal BMI. Participants with BMI ≥ 25 and < 30 kg/m2 were defined as overweight. Participants with BMI ≥ 30 kg/m2 were defined as obese. The study endpoint was all-cause and CV mortality. All-cause mortality was caused primarily by heart disease, malignant neoplasms, chronic lower respiratory disease, and cerebrovascular disease. CV mortality was caused by heart disease and cerebrovascular disease. Death data were extracted from the National Center for Health Statistics 2019 public-use linked mortality files.

### Covariates

All participants were measured for height, waist circumference (WC), weight, and systolic blood pressure (SBP) by trained examiners at the mobile examination center. Blood pressure was measured three times to obtain an average. BMI was calculated using the following formula BMI = body weight (kg)/height^2^ (m^2^). Fasting venous blood samples were collected for measurement of total cholesterol (TC, mmol/L), high-density lipoprotein cholesterol (HDL-C, mmol/L), serum creatinine (SCr, umol/L), glucose (mmol/L), and insulin (pmol/L) according to NHANES quality assurance and quality control protocols. Estimated glomerular filtration rate (eGFR) was calculated using the 4-variable MDRD equations [12]. Education level was categorized as less than high school, high school or equivalent, college or higher. Poverty ratio was calculated as the ratio of monthly family income to the poverty line and divided into 3 groups: <1.0, 1.0–2.0, and ≥ 3.0. Marital status was divided into married, never married and other marital status (widowed, divorced, separated and living with partner) [13]. Participants were classified as never smokers (smoked less than 100 cigarettes in lifetime), former smokers (smoked > 100 cigarettes in lifetime but did not smoke currently), and current smokers (average current smoking ≥ 1/day) [14]. There were five races, including Mexican American, other Hispanic, non-Hispanic white, non-Hispanic black, and other race. Hypertension was defined as self-reported history of hypertension or use of antihypertensive medication. Diabetes mellitus was defined as self-reported status of diabetes mellitus diagnosis, current use of hypoglycemic therapy, or glycosylated hemoglobin (HbA1c) level ≥ 6.5%, FPG level ≥ 7.0 mmol/L.

### Statistical analysis

Baseline characteristics are presented as weighted means and standard deviations (SDs) for continuous variables. Unweighted numbers and weighted proportions were also presented for categorical variables. The test for differences in categorical variables was the chi-squared test with Rao & Scott’s second order correction. The significant difference between the baseline data of the groups was determined by the t-test for data with normal distribution and by the Mann-Whitney U test for data with skewed distribution. Adjusted variables in multivariate Cox regression models were based on clinical correlation, and the final regression model was determined based on the number of events [15]. The results of COX regression analysis were expressed as hazard ratio (HR) and 95% confidence interval (CI). Restricted cubic spline was used to analyze the nonlinear association of HOMA2-IR with HR of all-cause mortality and CV mortality. Propensity score matching was performed at a ratio of 1:1:1 for statistical adjustment of original participant data in three groups. All statistical analysis for this study was completed by R (version 4.2.2, http://www.R-project.org) and STATA (version17). Two-sided *P* < 0.05 indicated statistically significant.

## Results

### Baseline characteristics

A total of 7,085 eligible participants aged 20 years or older were enrolled into the study cohort. The baseline characteristics of this diverse assembly were categorized based on distinct HOMA2-IR tertiles. The weighted mean age of the study participants was 45.14 ± 15.97 years, with females representing 49.30% of the weighted composition, for a total of 3,434 individuals. Within this cohort, a notable subset of 797 participants were diagnosed with diabetes mellitus, representing a weighted percentage of 7.85%. Compared with participants in tertile 2, those in tertile 3 had a higher prevalence of older age, fewer females, less non-Hispanic white ethnicity, less college education, and higher BMI, WC, SBP, HbA1C, FPG, and fasting insulin. In addition, a higher proportion of individuals in tertile 3 had a diagnosis of hypertension and diabetes mellitus. Notably, individuals in tertile 3 had increased use of antihypertensive, hypoglycemic, and lipid-lowering medications (Table 1).

**Table 1:** The demographic and clinical characteristics of study population by baseline HOMA2-IR.

Characteristics	Overall	HOMA2-IR			
		Tertile 1	Tertile 2	Tertile 3	*P* value
Participants	7085	2356	2351	2378	
Age(years)	45.14 ± 15.97	43.89 ± 15.70	44.73 ± 16.18	47.01 ± 15.86^#£^	< 0.001
Male, n (%)	3651(50.70)	1205(47.96)	1187(49.86)	1259(54.71) ^#£^	< 0.001
**Race, n (%)**					< 0.001
Non-Hispanic White	3407(70.99)	1306(76.11)	1145(70.94) ^*^	956(65.38) ^#£^	
Mexican American	1647(7.70)	416(5.77)	566(7.86)	665(9.67)	
Non-Hispanic Black	1477(11.14)	465(9.78)	430(9.61)	582(14.42)	
Other Hispanic	296(5.55)	69(3.12)	119(6.92)	108(6.66)	
Other Race	258(4.61)	100(5.21)	91(4.68)	67(3.88)	
**Education level, n (%)**					< 0.001
High school or equivalent	3580(56.29)	1178(52.94)	1192(57.41) ^*^	1210(58.68) ^#£^	
Less than high school	2238(20.16)	648(17.16)	736(19.74)	854(23.96)	
College or above	1267(23.56)	530(29.90)	423(22.85)	314(17.35)	
**Family income to poverty ratio, n (%)**					0.004
≥ 1&<3	2972(36.57)	939(34.92)	967(36.04)	1066(39.01) ^#^	
≥ 3	2889(51.19)	1018(54.62)	987(51.34)	884(47.21)	
< 1	1224(12.24)	399(10.46)	397(12.62)	428(13.78)	
**Marital status, n (%)**					0.875
Married	3970(59.81)	1288(59.50)	1331(60.35)	1351(59.51)	
Others	1952(23.64)	645(23.62)	641(22.90)	666(24.53)	
Never married	1163(16.55)	423(16.88)	379(16.75)	361(15.96)	
**Smoking status, n (%)**					0.002
Never	3624(49.94)	1175(49.24)	1205(49.59)	1244(51.11) ^#^	
Former	1806(24.90)	538(22.09)	608(25.42)	660(27.41)	
Current	1655(25.16)	643(28.67)	538(24.99)	474(21.48)	
BMI (kg/m^2^)	27.00(23.70,31.09)	23.88(21.58,26.47)	26.96(24.40,30.23) ^*^	31.50(28.01,36.01) ^#£^	< 0.001
WC (cm)	95.00(84.80,105.50)	85.50(77.60,93.50)	95.00(87.00,103.00) ^*^	106.70(97.60,116.30) ^#£^	< 0.001
SBP (mmHg)	119.33(110.00,132.00)	115.33(106.67,127.33)	118.67(110.00,131.33) ^*^	124.00(114.67,134.67) ^#£^	< 0.001
DBP (mmHg)	72.37 ± 10.98	70.54 ± 10.64	72.47 ± 10.46^*^	74.27 ± 11.60^#£^	< 0.001
Hypertension, n (%)	2200(25.92)	527(16.66)	685(23.88) ^*^	988(38.54) ^#£^	< 0.001
Diabetes, n (%)	797(7.85)	110(2.75)	181(4.92) ^*^	506(16.87) ^#£^	< 0.001
Antihypertensive drugs, n (%)	1537(18.17)	333(9.88)	478(16.50) ^*^	726(29.46) ^#£^	< 0.001
Glucose-lowering drugs, n (%)	426(3.86)	54(1.23)	98(2.40) ^*^	274(8.52) ^#£^	< 0.001
Lipid-lowering drugs, n (%)	840(12.11)	193(7.04)	282(11.82) ^*^	365(18.28) ^#£^	< 0.001
HbA1C (%)	5.30(5.10,5.50)	5.20(5.00,5.40)	5.22(5.00,5.50) ^*^	5.50(5.20,5.80) ^#£^	< 0.001
Glucose (mmol/L)	5.32(4.96,5.74)	5.07(4.79,5.38)	5.33(4.99,5.69) ^*^	5.66(5.26,6.26) ^#£^	< 0.001
Insulin(pmol/L)	54.30(36.83,84.90)	31.50(24.30,37.20)	55.93(48.84,65.34) ^*^	109.29(88.44,144.66) ^#£^	< 0.001
TC (mmol/L)	5.14 ± 1.06	5.02 ± 1.03	5.16 ± 1.04^*^	5.24 ± 1.10^#^	< 0.001
LDL-C (mmol/L)	3.15 ± 0.95	3.01 ± 0.91	3.21 ± 0.92^*^	3.25 ± 0.99^#^	< 0.001
HDL-C (mmol/L)	1.27(1.06,1.56)	1.45(1.22,1.76)	1.24(1.06,1.53) ^*^	1.11(0.95,1.32) ^#£^	< 0.001
TG (mmol/L)	1.21(0.82,1.80)	0.90(0.67,1.28)	1.24(0.86,1.71) ^*^	1.65(1.16,2.39) ^#£^	< 0.001
eGFR(ml/min/1.73m^2^)	100.84 ± 29.10	99.47 ± 26.25	101.61 ± 29.74	101.47 ± 31.22	0.178

*HOMA2-IR* homeostatic model assessment, *BMI* body mass index, *WC* waist circumference, *SBP* systolic blood pressure, *DBP* diastolic blood pressure, *HbA1C* glycosylated hemoglobin, *TC* total cholesterol, *LDL-C* low-density lipoprotein, *LDL-C* high-density lipoprotein, TG triglycerides, *eGFR* estimated glomerular filtration rate. Weighted means and standard deviation (SD) for continuous variables. Unweighted numbers and weighted proportions for categorical variables. *: *P* value between tertile1 and tertile2 < 0.05. #: *P* value between tertile1 and tertile3 < 0.05. £: *P* value between tertile2 and tertile3 < 0.05

### HOMA2-IR and mortality

Before multivariate-adjusted Cox regression analysis, we confirmed that all covariates met the proportional hazards assumption. In the first model (model 1), a discernible association between HOMA2-IR and all-cause mortality emerged. This association persisted even after extensive adjustment for a constellation of variables, including smoking status, educational attainment, family income, marital status, blood pressure, BMI, blood lipid levels, and other relevant factors. Specifically, HOMA2-IR remained statistically significant and positively associated with all-cause mortality (HR:1.15;95%CI:1.07–1.24). After HOMA2-IR was divided into three tertiles and included in the model, compared with the tertile1 reference in the fully adjusted model, the risk of all-cause mortality was significantly reduced in participants with obesity in tertile 2 (HR:0.68;95%CI:0.52–0.89) (Table 2). Notably, the initially observed association between HOMA2-IR and CV mortality lost statistical significance in the fully adjusted model (Table 3). In the stratified analysis, the significant association between HOMA2-IR and all-cause mortality was present in all subgroups except for male participants. There was a significant interaction between HOMA2-IR and age for all-cause mortality (*P* for interaction = 0.016) (Figure S1). In participants without diabetes, HOMA2-IR was still positively associated with all-cause mortality (HR:1.12;95%CI:1.03–1.22). Tertile 2 of HOMA2-IR showed decreased risk of all-cause mortality in participants without diabetes with obesity compared to tertile 1 (HR:0.72;95%CI:0.52–0.98) (Table S1). After we divided the participants into three tertiles based on WC, a decreased risk of all-cause mortality was observed in tertile 2 of HOMA2-IR among participants with the highest WC tertile (HR:0.74;95%CI:0.60–0.92) (Table S3). When we divided the study population into three tertiles based on the TG/HDL-C ratio, another indicator of insulin resistance, the risk of all-cause mortality remained significantly reduced in tertile 2 of HOMA2-IR among participants with the highest tertile of TG/HDL-C (HR:0.78;95%CI:0.61-1.00) (Table S5). Propensity score matching was used to adjust for demographic and clinical characteristics for the three groups (Table S7). Among propensity score matched participants, a similar reduction in all-cause mortality was observed in tertile 2 of participants with obesity (HR:0.58;95%CI:0.42–0.79) (Table S8). No similar result was found in the analysis for CV mortality (Table S2, 4, 6, 9).

**Table 2:** The associations of HOMA2-IR with all-cause mortality in study participants

HOMA2-IR	Events, n (%)	HR (95% CI), *P* value					
		Model 1		Model2		Model3	
**All-cause mortality** **All participants**							
Per SD	1666(23.5%)	1.21(1.13–1.29)	< 0.001	1.21(1.13–1.28)	< 0.001	1.15(1.07–1.24)	< 0.001
Tertile 1	519(22.0%)	Ref.		Ref.		Ref.	
Tertile 2	520(22.2%)	1.01(0.86–1.19)	0.890	1.03(0.88–1.22)	0.682	0.96(0.81–1.13)	0.606
Tertile 3	627(26.4%)	1.14(0.96–1.36)	0.133	1.17(0.99–1.39)	0.065	0.94(0.79–1.13)	0.540
*P* for trend			0.127		0.063		0.544
**BMI < 25 kg/m2**							
Per SD	506(22.6%)	1.16(1.02–1.31)	0.019	1.15(1.03–1.29)	0.014	1.14(1.04–1.26)	0.007
Tertile 1	159(21.5%)	Ref.		Ref.		Ref.	
Tertile 2	147(19.9%)	0.88(0.63–1.23)	0.452	0.91(0.64–1.29)	0.598	1.06(0.73–1.54)	0.745
Tertile 3	200(26.2%)	1.05(0.79–1.40)	0.749	1.07(0.79–1.46)	0.653	1.19(0.83–1.70)	0.337
*P* for trend			0.611		0.547		0.309
**25 ≤ BMI < 30 kg/m2**							
Per SD	621(24.4%)	1.18(1.08–1.28)	< 0.001	1.15(1.04–1.26)	0.006	1.12(1.00-1.25)	0.061
Tertile 1	184(22.1%)	Ref.		Ref.		Ref.	
Tertile 2	190(22.4%)	1.13(0.87–1.46)	0.366	1.07(0.84–1.36)	0.564	1.06(0.82–1.38)	0.639
Tertile 3	247(28.6%)	1.13(0.88–1.46)	0.326	1.08(0.86–1.37)	0.511	0.97(0.77–1.23)	0.818
*P* for trend			0.350		0.529		0.795
**BMI ≥ 30 kg/m2**							
Per SD	539(23.5%)	1.27(1.14–1.42)	< 0.001	1.26(1.14–1.38)	< 0.001	1.12(1.01–1.25)	0.031
Tertile 1	174(22.9%)	Ref.		Ref.		Ref.	
Tertile 2	153(19.9%)	0.75(0.56-1.00)	0.048	0.73(0.56–0.96)	0.024	0.68(0.52–0.89)	0.005
Tertile 3	212(27.6%)	1.30(0.99–1.73)	0.064	1.27(0.97–1.66)	0.080	0.92(0.71–1.20)	0.538
*P* for trend			0.052		0.065		0.645

Model 1 was adjusted for age, gender, race; Model 2 was adjusted for age, gender, race, smoking status, education level, family income to poverty ratio, marital status; Model 3was adjusted for age, gender, race, smoking status, education level, family income to poverty ratio, marital status, hypertension, diabetes, BMI, SBP, LDL-C, HDL-C, TG, HbA1c, eGFR

**Table 3:** The associations of HOMA2-IR with CV mortality in study participants

HOMA2-IR	Events, n (%)	HR (95% CI), *P* value					
		Model 1		Model2		Model3	
**Cardiovascular mortality** **All participants**							
Per SD	555(7.8%)	1.24(1.10–1.40)	< 0.001	1.24(1.11–1.39)	< 0.001	1.12(0.97–1.31)	0.130
Tertile 1	172(7.3%)	Ref.		Ref.		Ref.	
Tertile 2	166(7.1%)	0.95(0.74–1.22)	0.691	0.98(0.76–1.26)	0.859	0.86(0.65–1.13)	0.268
Tertile 3	217(9.1%)	1.22(0.92–1.61)	0.165	1.26(0.96–1.66)	0.098	0.91(0.63–1.30)	0.598
*P* for trend			0.147		0.089		0.621
**BMI < 25 kg/m2**							
Per SD	148(6.6%)	1.17(0.96–1.43)	0.128	1.18(0.96–1.45)	0.121	1.15(1.00-1.33)	0.056
Tertile 1	47(6.3%)	Ref.		Ref.		Ref.	
Tertile 2	40(5.4%)	1.12(0.64–1.95)	0.702	1.12(0.61–2.05)	0.709	1.50(0.82–2.75)	0.189
Tertile 3	61(8.0%)	1.21(0.82–1.79)	0.343	1.20(0.79–1.83)	0.399	1.36(0.86–2.15)	0.195
*P* for trend			0.359		0.417		0.244
**25 ≤ BMI < 30 kg/m2**							
Per SD	207(8.1%)	1.15(1.00-1.33)	0.053	1.11(0.97–1.28)	0.142	1.09(0.94–1.26)	0.252
Tertile 1	63(7.6%)	Ref.		Ref.		Ref.	
Tertile 2	63(7.4%)	0.96(0.65–1.41)	0.825	0.91(0.61–1.34)	0.619	0.89(0.59–1.34)	0.573
Tertile 3	81(9.4%)	0.99(0.66–1.49)	0.977	0.95(0.64–1.41)	0.785	0.85(0.54–1.34)	0.491
*P* for trend			0.992		0.813		0.496
**BMI ≥ 30 kg/m2**							
Per SD	200(8.7%)	1.25(1.02–1.53)	0.030	1.24(1.03–1.48)	0.022	1.06(0.85–1.32)	0.624
Tertile 1	74(9.8%)	Ref.		Ref.		Ref.	
Tertile 2	47(6.1%)	0.66(0.41–1.06)	0.084	0.65(0.42-1.00)	0.052	0.64(0.39–1.05)	0.078
Tertile 3	79(10.3%)	1.19(0.78–1.83)	0.418	1.20(0.80–1.82)	0.379	0.86(0.53–1.42)	0.566
*P* for trend			0.385		0.350		0.611

Model 1 was adjusted for age, gender, race; Model 2 was adjusted for age, gender, race, smoking status, education level, family income to poverty ratio, marital status; Model 3was adjusted for age, gender, race, smoking status, education level, family income to poverty ratio, marital status, hypertension, diabetes, BMI, SBP, LDL-C, HDL-C, TG, HbA1c, eGFR

### Non-linear relationships in obese participants

**Fig. 2:**
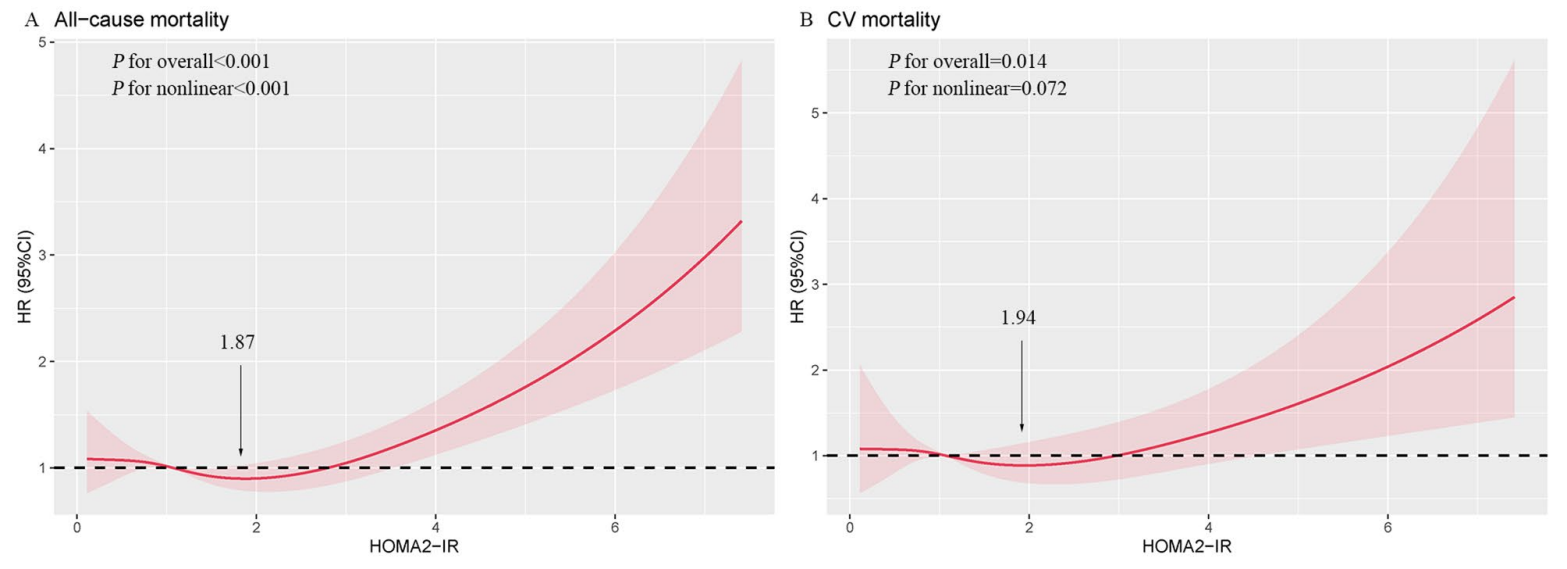
Multivariable-adjusted hazard ratios for (**A**) all-cause and (**B**) CV mortality in general population based on restricted cubic spines for HOMA2-IR. Adjusted model included age, gender, race, smoking status, education level, family income to poverty ratio, marital status, hypertension, diabetes, BMI, SBP, LDL-C, HDL-C, TG, HbA1c, eGFR.

**Fig. 3:**
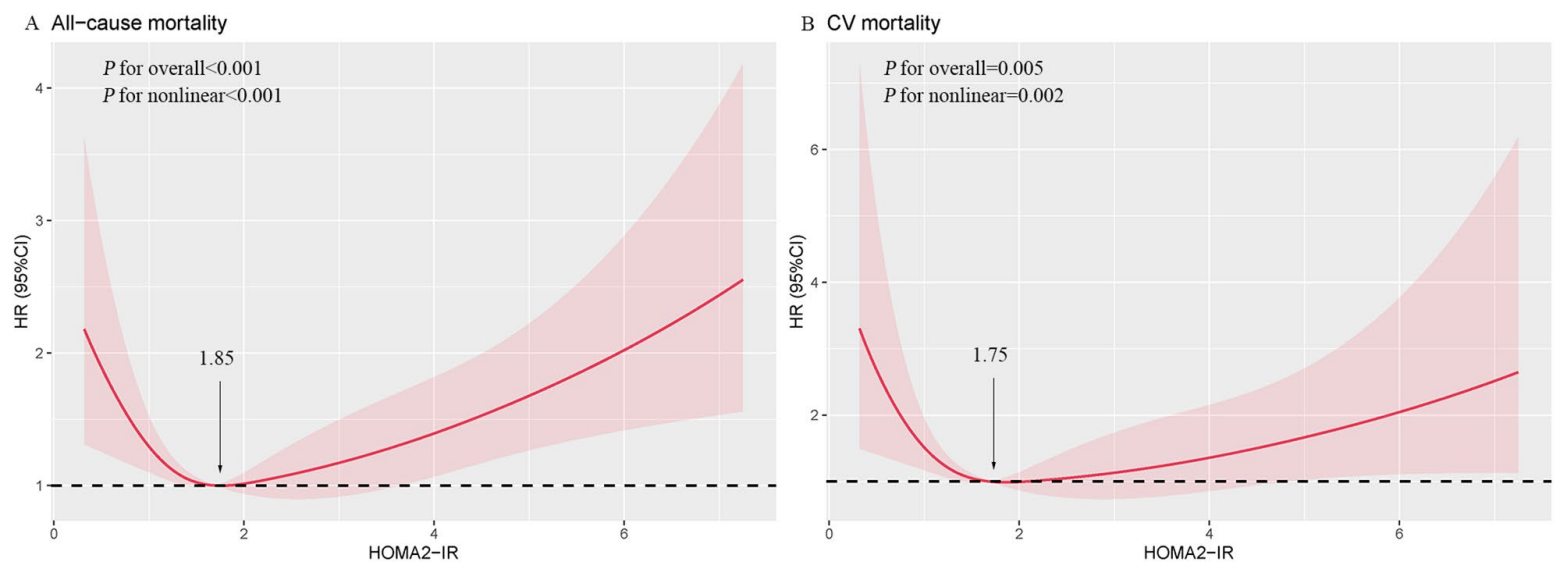
Multivariable-adjusted hazard ratios for (**A**) all-cause and (**B**) CV mortality among participants with obesity based on restricted cubic spines for HOMA2-IR. Adjusted model included age, gender, race, smoking status, education level, family income to poverty ratio, marital status, hypertension, diabetes, BMI, SBP, LDL-C, HDL-C, TG, HbA1c, eGFR.

In the fully adjusted model, restricted cubic spline unraveled a nonlinear J-shaped association between HOMA2-IR and all-cause mortality (*P* for nonlinearity < 0.001). This intricate pattern revealed that the risk of all-cause mortality showed relative stability until a threshold around 1.87 of HOMA2-IR was reached, after which it began to rise. A linear association was observed between HOMA2-IR and CV mortality (*P* for nonlinearity = 0.072) (Fig. 2). Participants were then divided into three groups based on BMI. The strong U-shaped relationship of HOMA2-IR with all-cause mortality in participants with obesity indicated a substantial risk reduction before a threshold of 1.85 of HOMA2-IR, followed by a subsequent increase (*P* for nonlinearity < 0.001). In the lower range of HOMA2-IR in participants with obesity, a gradual reduction in CV mortality risk was observed, extending to approximately 1.75 (*P* for nonlinearity = 0.002) (Fig. 3).

## Discussion

In our study, we found a remarkable positive association between HOMA2-IR and all-cause mortality. In addition, we categorized HOMA2-IR into three tertiles. Of particular importance, our investigation revealed a distinct, non-linear, U-shaped association between HOMA2-IR and both all-cause and CV mortality in the obese population. This intricate pattern delineated a unique trajectory in which mortality risk gradually decreased before reaching a nadir and subsequently increased, providing a nuanced perspective on the complex relationship between insulin resistance and mortality outcomes in the context of obesity. To the best of our knowledge, this is the first study to evaluate the nonlinear correlations of HOMA2-IR with mortality in the obese population.

Many previous studies have shown that elevated levels of insulin resistance were positively associated with all-cause and CV mortality in both general and specific populations [16, 17]. In young and middle-aged Chinese, participants who progressed to T2D had higher HOMA2-IR, an indicator of insulin resistance, while T2DM patients with glycemic worsening had higher HOMA2-IR and lower HOMA2-B [18]. A positive association between HOMA2-IR and all-cause mortality was observed in our study, which is similar to previous studies. Higher levels of insulin resistance index were significantly associated with reduced eGFR and chronic kidney disease [19, 20]. Levels of estimated glucose disposal rate (eGDR) were independently associated with micro- or macroalbuminuria, the albuminuric diabetic kidney disease (DKD) phenotypes, eGFR, and the non-albuminuric DKD phenotype [21]. In patients with obesity, the level of insulin resistance correlated with nonalcoholic steatohepatitis [22]. In females with breast cancer, elevated HOMA scores were associated with increased cancer-cause all-cause mortality after adjustment for potential confounders [23]. Endogenous insulin receptor/insulin-like growth factor-I receptor/Akt may mediate the effects in promoting tumorigenesis and progression in animal models of insulin resistance [24]. The urinary system, nervous system, motor system, digestive system and tumor are all affected by insulin resistance and serum glucose fluctuations, which may explain the stronger significance of HOMA2-IR for all-cause mortality than CV mortality. However, no significant association between HOMA2-IR and CV mortality was observed in our study. There were significant differences in the diagnostic yield of cardiometabolic diseases among different tertiles of HOMA2-IR, as well as the use of antihypertensive, hypoglycemic, and lipid-lowering medications, which may affect the predictive power of HOMA2-IR for CV mortality [25].

Our study showed a nonlinear U-shaped association between HOMA2-IR and all-cause mortality in the population with obesity. Compared with tertile 1 of HOMA2-IR, tertile2 and tertile 3 did not show a significantly elevated adjusted hazard ratio for all-cause mortality. A previous study suggested that HOMA-IR was significantly associated with all-cause mortality only in participants with a BMI < 25.2 kg/m^2^, but not in those with a BMI ≥ 25.2 kg/m^2^ [26]. In individuals without obesity, IR may be caused by factors other than BMI, whereas in individuals with IR associated with high BMI, IR did not confer an independent additional risk of mortality. In our study, the comparison between tertile 3 and tertile 2 did not show an increased risk of all-cause mortality in individuals with obesity. This observation may be due to the fact that the IR in the obese population was mainly due to increased BMI. The triglyceride glucose index is an indicator of insulin resistance, and the first and second quartiles showed the increased risk of all-cause mortality compared with the third quartile of the triglyceride glucose index [27]. This finding is consistent with our study.

We found that excessively reduced levels of insulin resistance were correlated with increased mortality in individuals with obesity. We proposed several potential mechanisms to explain our findings. Notably, lower IR levels were associated with lower fasting glucose levels [28, 29]. The increase in epinephrine caused by repeated hypoglycemia promoted endometrial thickening and smooth muscle cell proliferation in Goto-Kakizaki rats, and glucose injection could inhibit hypoglycemia and abolish endometrial thickening [30]. The U-shaped curve suggested that both hypoglycemia and IR, represented by low and high HOMA2-IR, may lead to adverse health outcomes. Clinicians can benefit specific groups in population with obesity by targeting interventions to improve metabolic balance.

There are several drawbacks to this study. First, we included only a subset of the population, and it could not be verified in the unselected population because of some missing insulin data. In addition, the mechanism of the relationship between HOMA2-IR and mortality in people with obesity could not be confirmed in this study and needs to be explored by subsequent animal experiments. Finally, HOMA2-IR may not reflect long-term levels of insulin resistance when calculated from a single measurement of plasma glucose and insulin concentrations.

## Conclusions

HOMA2-IR stratification was associated with all-cause mortality. Excessively low levels of IR were correlated with increased mortality in individuals with obesity. It may be worth noting that maintaining these levels above excessively low thresholds could potentially be beneficial in improving the well-being of populations with obesity.

### Electronic supplementary material

Below is the link to the electronic supplementary material.

Supplementary Material 1

Supplementary Material 2

## Data Availability

This study analyzed publicly available datasets. This data is available on NHANES’s official website ( https://www.cdc.gov/nchs/nhanes/index.htm).

## Abbreviations

HOMA2-IR: Homeostatic model assessment
CV: Cardiovascular
IR: Insulin resistance
T2DM: Type 2 diabetes mellitus
MS: Metabolic syndrome
FPG: Fasting plasma glucose
BMI: Body mass index
NHANES: National Health and Nutrition Examination Survey
WC: Waist circumference
TC: Total cholesterol
SCr: Serum creatinine
LDL-C: Low-density lipoprotein
eGFR: Estimated glomerular filtration rate
HbA1c: Glycosylated hemoglobin
CI: Confidence intervals
HR: Hazard ratio

## References

[1] Fahed G, Aoun L, Bou Zerdan M, Allam S, Bou Zerdan M, Bouferraa Y, et al. Metabolic syndrome: updates on pathophysiology and management in 2021. Int J Mol Sci. 2022;23(2). 10.3390/ijms23020786. 10.3390/ijms23020786 PMC8775991 35054972

[2] Herningtyas EH, Ng TS (2019). Prevalence and distribution of metabolic syndrome and its components among provinces and ethnic groups in Indonesia. BMC Public Health.

[3] Caumo A, Perseghin G, Brunani A, Luzi L (2006). New insights on the simultaneous assessment of insulin sensitivity and beta-cell function with the HOMA2 method. Diabetes Care.

[4] Song YS, Hwang YC, Ahn HY, Park CY (2016). Comparison of the usefulness of the updated Homeostasis Model Assessment (HOMA2) with the original HOMA1 in the prediction of type 2 diabetes Mellitus in koreans. Diabetes Metab J.

[5] Levy JC, Matthews DR, Hermans MP (1998). Correct homeostasis model assessment (HOMA) evaluation uses the computer program. Diabetes Care.

[6] Geloneze B, Vasques AC, Stabe CF, Pareja JC, Rosado LE, Queiroz EC (2009). HOMA1-IR and HOMA2-IR indexes in identifying insulin resistance and metabolic syndrome: Brazilian metabolic syndrome study (BRAMS). Arq Bras Endocrinol Metabol.

[7] Safar FH, Mojiminiyi OA, Al-Rumaih HM, Diejomaoh MF (2011). Computational methods are significant determinants of the associations and definitions of insulin resistance using the homeostasis model assessment in women of reproductive age. Clin Chem.

[8] Ahmed B, Sultana R, Greene MW (2021). Adipose tissue and insulin resistance in obese. Biomed Pharmacother.

[9] Hinnouho GM, Czernichow S, Dugravot A, Batty GD, Kivimaki M, Singh-Manoux A (2013). Metabolically healthy obesity and risk of mortality: does the definition of metabolic health matter?. Diabetes Care.

[10] Hong S, Han K, Park CY (2020). The triglyceride glucose index is a simple and low-cost marker associated with atherosclerotic cardiovascular disease: a population-based study. BMC Med.

[11] von Elm E, Altman DG, Egger M, Pocock SJ, Gotzsche PC, Vandenbroucke JP (2007). The strengthening the reporting of Observational studies in Epidemiology (STROBE) statement: guidelines for reporting observational studies. Lancet.

[12] Poggio ED, Nef PC, Wang X, Greene T, Van Lente F, Dennis VW (2005). Performance of the Cockcroft-Gault and modification of diet in renal disease equations in estimating GFR in ill hospitalized patients. Am J Kidney Dis.

[13] Iranpour S, Sabour S, Koohi F, Saadati HM,.S. The trend and pattern of depression prevalence in the U. J Affect Disord. 2022;298(Pt A):508–15. 10.1016/j.jad.2021.11.027. Data from National Health and Nutrition Examination Survey (NHANES) 2005 to 2016. 10.1016/j.jad.2021.11.027 34785265

[14] Wang J, Liu F, Kong R, Han X (2022). Association between Globulin and Diabetic Nephropathy in Type2 Diabetes Mellitus patients: a cross-sectional study. Front Endocrinol (Lausanne).

[15] Stone GW, Maehara A, Lansky AJ, de Bruyne B, Cristea E, Mintz GS (2011). A prospective natural-history study of coronary atherosclerosis. N Engl J Med.

[16] Chen J, Wu K, Lin Y, Huang M, Xie S (2023). Association of triglyceride glucose index with all-cause and cardiovascular mortality in the general population. Cardiovasc Diabetol.

[17] Pang J, Qian L, Che X, Lv P, Xu Q. TyG index is a predictor of all-cause mortality during the long-term follow-up in middle-aged and elderly with hypertension. Clinical and experimental hypertension (New York, NY: 1993). 2023;45(1):2272581.10.1080/10641963.2023.2272581. 10.1080/10641963.2023.2272581 37902269

[18] Fan B, Wu H, Shi M, Yang A, Lau ESH, Tam CHT (2022). Associations of the HOMA2-%B and HOMA2-IR with progression to diabetes and glycaemic deterioration in young and middle-aged Chinese. Diabetes Metab Res Rev.

[19] Fu X, Xu Z, Tan Q, Wei W, Wang Z (2023). Association between a high triglyceride-glucose index and chronic kidney disease in adult patients with latent autoimmune diabetes. BMC Endocr Disorders.

[20] Cui C, Liu L, Zhang T, Fang L, Mo Z, Qi Y (2023). Triglyceride-glucose index, renal function and cardiovascular disease: a national cohort study. Cardiovasc Diabetol.

[21] Penno G, Solini A, Orsi E, Bonora E, Fondelli C, Trevisan R (2021). Insulin resistance, diabetic kidney disease, and all-cause mortality in individuals with type 2 diabetes: a prospective cohort study. BMC Med.

[22] Rivière B, Jaussent A, Macioce V, Faure S, Builles N, Lefebvre P (2022). The triglycerides and glucose (TyG) index: a new marker associated with nonalcoholic steatohepatitis (NASH) in obese patients. Diabetes Metab.

[23] Duggan C, Irwin ML, Xiao L, Henderson KD, Smith AW, Baumgartner RN (2011). Associations of insulin resistance and adiponectin with mortality in women with breast cancer. J Clin Oncol.

[24] Novosyadlyy R, Lann DE, Vijayakumar A, Rowzee A, Lazzarino DA, Fierz Y (2010). Insulin-mediated acceleration of breast cancer development and progression in a nonobese model of type 2 diabetes. Cancer Res.

[25] Li H, Jiang Y, Su X, Meng Z (2023). The triglyceride glucose index was U-shape associated with all-cause mortality in population with cardiovascular diseases. Diabetol Metab Syndr.

[26] Ausk KJ, Boyko EJ, Ioannou GN (2010). Insulin resistance predicts mortality in nondiabetic individuals in the U.S. Diabetes Care.

[27] Du L, Xu X, Wu Y, Yao H. Association between the triglyceride glucose index and cardiovascular mortality in obese population. Nutrition, metabolism, and cardiovascular diseases: NMCD. 2024;34(1):107–11.10.1016/j.numecd.2023.08.007. 10.1016/j.numecd.2023.08.007 37949711

[28] Park C, Guallar E, Linton JA, Lee DC, Jang Y, Son DK (2013). Fasting glucose level and the risk of incident atherosclerotic cardiovascular diseases. Diabetes Care.

[29] Emerging Risk Factors C, Sarwar N, Gao P, Seshasai SR, Gobin R, Kaptoge S (2010). Diabetes mellitus, fasting blood glucose concentration, and risk of vascular disease: a collaborative meta-analysis of 102 prospective studies. Lancet.

[30] Yasunari E, Mita T, Osonoi Y, Azuma K, Goto H, Ohmura C (2014). Repetitive hypoglycemia increases circulating adrenaline level with resultant worsening of intimal thickening after vascular injury in male goto-kakizaki rat carotid artery. Endocrinology.

