# Supplementary figures and images for: Non-linear associations of HOMA2-IR with all-cause mortality in general populations: insights from NHANES 1999–2006
Source: BMC Public Health. 2024 Feb 22;24:574. doi: 10.1186/s12889-024-18026-7 (PMC10885457; doi:10.1186/s12889-024-18026-7)

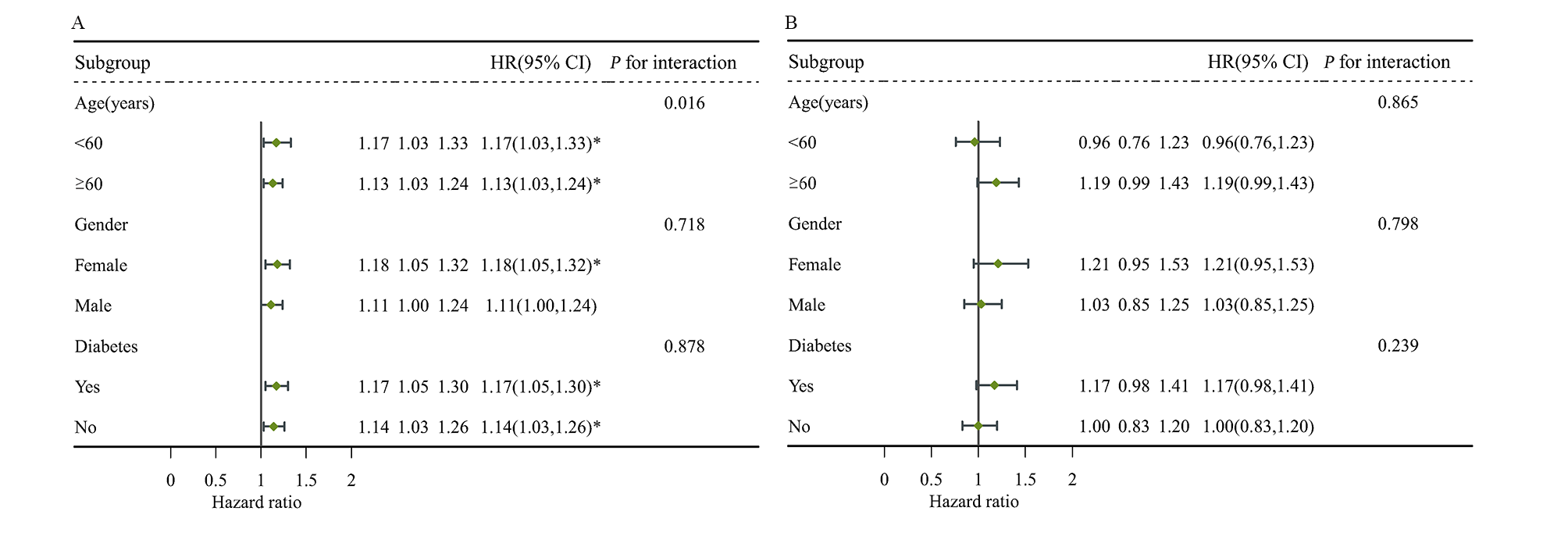

Supplement: Supplementary file 1 — Supplementary Material 1 [file 12889_2024_18026_MOESM1_ESM.tif]
