# Supplementary material for: Non-linear associations of HOMA2-IR with all-cause mortality in general populations: insights from NHANES 1999–2006
Source: BMC Public Health. 2024 Feb 22;24:574. doi: 10.1186/s12889-024-18026-7 (PMC10885457; doi:10.1186/s12889-024-18026-7)
Supplement: Supplementary file 2 — Supplementary Material 2 [file 12889_2024_18026_MOESM2_ESM.docx]

**Table S1** The associations of HOMA2-IR with all-cause mortality among participants without diabetes.

| HOMA2-IR | Events, n (%) | HR (95% CI), *P* value | | | | | |
| --- | --- | --- | --- | --- | --- | --- | --- |
|  |  | Model 1 | | Model2 | | Model3 | |
| **All-cause mortality**  **All participants** | |  |  |  |  |  |  |
| Per SD | 1281(%) | 1.12(1.02-1.22) | 0.014 | 1.11(1.02-1.21) | 0.011 | 1.12(1.03-1.22) | 0.010 |
| Tertile 1 | 428(20.9%) | Ref. |  | Ref. |  | Ref. |  |
| Tertile 2 | 434(20.4%) | 0.93(0.77-1.12) | 0.451 | 0.93(0.78-1.12) | 0.461 | 0.94(0.78-1.14) | 0.539 |
| Tertile 3 | 419(19.9%) | 1.05(0.89-1.24) | 0.585 | 1.03(0.86-1.22) | 0.780 | 0.97(0.82-1.15) | 0.753 |
| *P* for trend |  |  | 0.558 |  | 0.753 |  | 0.769 |
| **BMI<25kg/m2** | |  |  |  |  |  |  |
| Per SD | 438(20.7%) | 1.04(0.94-1.14) | 0.484 | 1.05(0.95-1.16) | 0.320 | 1.08(0.99-1.19) | 0.100 |
| Tertile 1 | 140(20.3%) | Ref. |  | Ref. |  | Ref. |  |
| Tertile 2 | 139(19.3%) | 0.86(0.58-1.27) | 0.445 | 0.84(0.56-1.27) | 0.414 | 0.96(0.64-1.44) | 0.841 |
| Tertile 3 | 159(22.5%) | 0.98(0.69-1.39) | 0.905 | 0.99(0.69-1.41) | 0.944 | 1.14(0.80-1.61) | 0.473 |
| *P* for trend |  |  | 0.989 |  | 0.933 |  | 0.396 |
| **25≤BMI<30kg/m2** |  |  |  |  |  |  |  |
| Per SD | 484(21.2%) | 1.08(0.96-1.22) | 0.184 | 1.04(0.92-1.19) | 0.521 | 1.01(0.90-1.14) | 0.831 |
| Tertile 1 | 160(21.2%) | Ref. |  | Ref. |  | Ref. |  |
| Tertile 2 | 159(20.9%) | 1.05(0.76-1.45) | 0.758 | 1.04(0.77-1.40) | 0.820 | 1.04(0.76-1.42) | 0.827 |
| Tertile 3 | 165(21.5%) | 0.96(0.71-1.30) | 0.792 | 0.92(0.68-1.26) | 0.607 | 0.84(0.62-1.13) | 0.251 |
| *P* for trend |  |  | 0.766 |  | 0.583 |  | 0.231 |
| **BMI≥30kg/m2** |  |  |  |  |  |  |  |
| Per SD | 359(19.0%) | 1.23(1.06-1.43) | 0.007 | 1.21(1.05-1.40) | 0.009 | 1.15(0.99-1.33) | 0.071 |
| Tertile 1 | 125(20.1%) | Ref. |  | Ref. |  | Ref. |  |
| Tertile 2 | 105(16.6%) | 0.78(0.56-1.09) | 0.151 | 0.78(0.56-1.07) | 0.125 | 0.72(0.52-0.98) | 0.040 |
| Tertile 3 | 129(20.4%) | 1.13(0.81-1.58) | 0.482 | 1.10(0.78-1.55) | 0.590 | 0.86(0.60-1.23) | 0.409 |
| *P* for trend |  |  | 0.481 |  | 0.585 |  | 0.439 |

Model 1 was adjusted for age, gender, race; Model 2 was adjusted for age, gender, race, smoking status, education level, family income to poverty ratio, marital status; Model 3was adjusted for age, gender, race, smoking status, education level, family income to poverty ratio, marital status, hypertension, BMI, SBP, LDL-C, HDL-C, TG, HbA1c, eGFR.

**Table S2** The associations of HOMA2-IR with CV mortality among participants without diabetes.

| HOMA2-IR | Events, n (%) | HR (95% CI), *P* value | | | | | |
| --- | --- | --- | --- | --- | --- | --- | --- |
|  |  | Model 1 | | Model2 | | Model3 | |
| **Cardiovascular mortality**  **All participants** | |  |  |  |  |  |  |
| Per SD | 417(6.6%) | 1.10(0.96-1.25) | 0.164 | 1.10(0.97-1.24) | 0.141 | 1.00(0.85-1.17) | 0.998 |
| Tertile 1 | 137(6.7%) | Ref. |  | Ref. |  | Ref. |  |
| Tertile 2 | 144(6.8%) | 0.86(0.63-1.18) | 0.346 | 0.87(0.64-1.18) | 0.376 | 0.92(0.67-1.26) | 0.594 |
| Tertile 3 | 136(6.4%) | 0.90(0.66-1.22) | 0.482 | 0.88(0.65-1.19) | 0.400 | 0.89(0.65-1.21) | 0.444 |
| *P* for trend |  |  | 0.499 |  | 0.414 |  | 0.449 |
| **BMI<25kg/m2** | |  |  |  |  |  |  |
| Per SD | 119(5.6%) | 0.99(0.82-1.19) | 0.879 | 1.00(0.83-1.22) | 0.974 | 1.05(0.88-1.26) | 0.576 |
| Tertile 1 | 40(5.8%) | Ref. |  | Ref. |  | Ref. |  |
| Tertile 2 | 38(5.3%) | 1.09(0.58-2.06) | 0.786 | 1.10(0.54-2.24) | 0.786 | 1.42(0.71-2.81) | 0.320 |
| Tertile 3 | 41(5.8%) | 1.01(0.63-1.61) | 0.971 | 0.99(0.60-1.64) | 0.978 | 1.29(0.80-2.08) | 0.295 |
| *P* for trend |  |  | 0.969 |  | 0.906 |  | 0.346 |
| **25≤BMI<30kg/m2** |  |  |  |  |  |  |  |
| Per SD | 166(7.3%) | 1.12(0.92-1.36) | 0.273 | 1.07(0.87-1.30) | 0.527 | 1.05(0.84-1.32) | 0.644 |
| Tertile 1 | 54(7.2%) | Ref. |  | Ref. |  | Ref. |  |
| Tertile 2 | 53(7.0%) | 0.91(0.59-1.40) | 0.682 | 0.89(0.57-1.39) | 0.607 | 0.85(0.52-1.39) | 0.524 |
| Tertile 3 | 59(7.7%) | 0.89(0.58-1.39) | 0.619 | 0.85(0.54-1.33) | 0.469 | 0.77(0.47-1.26) | 0.303 |
| *P* for trend |  |  | 0.625 |  | 0.476 |  | 0.306 |
| **BMI≥30kg/m2** |  |  |  |  |  |  |  |
| Per SD | 132(7.0%) | 0.99(0.73-1.36) | 0.959 | 1.00(0.74-1.35) | 0.998 | 0.84(0.61-1.15) | 0.281 |
| Tertile 1 | 58(9.3%) | Ref. |  | Ref. |  | Ref. |  |
| Tertile 2 | 32(5.1%) | 0.63(0.36-1.10) | 0.101 | 0.63(0.37-1.07) | 0.085 | 0.59(0.32-1.07) | 0.082 |
| Tertile 3 | 42(6.7%) | 0.89(0.54-1.45) | 0.630 | 0.90(0.55-1.46) | 0.658 | 0.63(0.36-1.12) | 0.115 |
| *P* for trend |  |  | 0.385 |  | 0.350 |  | 0.611 |

Model 1 was adjusted for age, gender, race; Model 2 was adjusted for age, gender, race, smoking status, education level, family income to poverty ratio, marital status; Model 3was adjusted for age, gender, race, smoking status, education level, family income to poverty ratio, marital status, hypertension, BMI, SBP, LDL-C, HDL-C, TG, HbA1c, eGFR.

**Table S3** The associations of HOMA2-IR with all-cause mortality among participants with different tertiles of WC.

| WC | HOMA2-IR | | | | | | | | *P* for trend |
| --- | --- | --- | --- | --- | --- | --- | --- | --- | --- |
|  | Tertile 1 | | Tertile 2 | | Tertile 3 | | Per SD |  |  |
| Tertile 1 | Ref. |  | 1.25(0.83-1.86) | 0.283 | 1.18(0.80-1.74) | 0.408 | 1.08(0.98-1.20) | 0.118 | 0.406 |
| Tertile 2 | Ref. |  | 1.17(0.91-1.50) | 0.215 | 0.95(0.70-1.29) | 0.758 | 1.04(0.93-1.17) | 0.504 | 0.828 |
| Tertile 3 | Ref. |  | 0.74(0.60-0.92) | 0.007 | 1.01(0.78-1.32) | 0.926 | 1.19(1.05-1.34) | 0.005 | 0.943 |

Statistical model was adjusted for age, gender, race, smoking status, education level, family income to poverty ratio, marital status, hypertension, diabetes, BMI, SBP, LDL-C, HDL-C, TG, HbA1c, eGFR.

**Table S4** The associations of HOMA2-IR with CV mortality among participants with different tertiles of WC.

| WC | HOMA2-IR | | | | | | | | *P* for trend |
| --- | --- | --- | --- | --- | --- | --- | --- | --- | --- |
|  | Tertile 1 | | Tertile 2 | | Tertile 3 | | Per SD |  |  |
| Tertile 1 | Ref. |  | 1.44(0.82-2.53) | 0.199 | 1.21(0.73-1.99) | 0.462 | 1.17(1.00-1.37) | 0.044 | 0.539 |
| Tertile 2 | Ref. |  | 1.00(0.63-1.60) | 0.983 | 0.87(0.48-1.58) | 0.652 | 1.04(0.84-1.29) | 0.708 | 0.672 |
| Tertile 3 | Ref. |  | 0.75(0.49-1.15) | 0.188 | 0.93(0.55-1.57) | 0.773 | 1.12(0.89-1.42) | 0.329 | 0.740 |

Statistical model was adjusted for age, gender, race, smoking status, education level, family income to poverty ratio, marital status, hypertension, diabetes, BMI, SBP, LDL-C, HDL-C, TG, HbA1c, eGFR.

**Table S5** The associations of HOMA2-IR with all-cause mortality among participants with different tertiles of TG/HDL-C ratio.

| TG/HDL | HOMA2-IR | | | | | | | | *P* for trend |
| --- | --- | --- | --- | --- | --- | --- | --- | --- | --- |
|  | Tertile 1 | | Tertile 2 | | Tertile 3 | | Per SD |  |  |
| Tertile 1 | Ref. |  | 1.06(0.78-1.44) | 0.698 | 1.02(0.67-1.56) | 0.922 | 1.15(1.01-1.31) | 0.035 | 0.913 |
| Tertile 2 | Ref. |  | 0.87(0.67-1.14) | 0.308 | 0.90(0.68-1.20) | 0.487 | 1.15(0.98,1.34) | 0.085 | 0.462 |
| Tertile 3 | Ref. |  | 0.78(0.61-1.00) | 0.049 | 0.98(0.70-1.36) | 0.887 | 1.13(1.01,1.28) | 0.041 | 0.889 |

Statistical model was adjusted for age, gender, race, smoking status, education level, family income to poverty ratio, marital status, hypertension, diabetes, BMI, SBP, LDL-C, HDL-C, TG, HbA1c, eGFR.

**Table S6** The associations of HOMA2-IR with CV mortality among participants with different tertiles of TG/HDL-C ratio.

| TG/HDL | HOMA2-IR | | | | | | | | *P* for trend |
| --- | --- | --- | --- | --- | --- | --- | --- | --- | --- |
|  | Tertile 1 | | Tertile 2 | | Tertile 3 | | Per SD |  |  |
| Tertile 1 | Ref. |  | 1.36(0.90-2.06) | 0.143 | 0.94(0.49-1.83) | 0.860 | 1.03(0.81,1.32) | 0.784 | 0.848 |
| Tertile 2 | Ref. |  | 0.88(0.56-1.38) | 0.573 | 0.73(0.47-1.15) | 0.178 | 1.13(0.85,1.51) | 0.398 | 0.176 |
| Tertile 3 | Ref. |  | 0.78(0.45-1.35) | 0.372 | 1.01(0.62-1.64) | 0.974 | 1.08(0.87,1.35) | 0.471 | 0.921 |

Statistical model was adjusted for age, gender, race, smoking status, education level, family income to poverty ratio, marital status, hypertension, diabetes, BMI, SBP, LDL-C, HDL-C, TG, HbA1c, eGFR.

**Table S7** The demographic and clinical characteristics of study population of propensity score matching.

| Characteristics | Overall | HOMA2-IR | | |  |
| --- | --- | --- | --- | --- | --- |
|  |  | Tertile 1 | Tertile 2 | Tertile 3 | *P* value |
| Participants | 3354 | 1118 | 1118 | 1118 |  |
| Age(years) | 43.93±15.24 | 44.27±15.97 | 44.10±16.11 | 43.46±13.41 | 0.934 |
| Male, n (%) | 1741(51.70) | 593(48.91) | 560(50.19) | 588(55.67) ^#£^ | 0.010 |
| **Race, n (%)** |  |  |  |  | <0.001 |
| Non-Hispanic White | 1578(68.53) | 610(73.68) | 537(70.29) ^*^ | 431(62.55) ^#£^ |  |
| Mexican American | 774(7.67) | 199(6.76) | 286(7.36) | 289(8.73) |  |
| Non-Hispanic Black | 675(10.94) | 216(9.50) | 182(9.11) | 277(14.39) |  |
| Other Hispanic | 191(7.84) | 34(3.15) | 78(9.11) | 79(9.45) |  |
| Other Race | 136(5.02) | 59(6.91) | 35(4.13) | 42(4.89) |  |
| **Education level, n (%)** |  |  |  |  | 0.038 |
| High school or equivalent | 1698(56.61) | 570(55.18) | 538(56.94) | 590(57.16) ^#^ |  |
| Less than high school | 1029(20.37) | 304(16.63) | 379(20.78) | 346(22.44) |  |
| College or above | 627(23.02) | 244(28.19) | 201(22.28) | 182(20.40) |  |
| **Family income to poverty ratio, n (%)** |  |  |  |  | 0.931 |
| ≥3 | 1397(51.22) | 452(52.68) | 477(51.04) | 468(50.43) |  |
| ≥1&<3 | 1340(35.32) | 446(34.44) | 436(35.28) | 458(36.01) |  |
| <1 | 617(13.46) | 220(12.88) | 205(13.68) | 192(13.56) |  |
| **Marital status, n (%)** |  |  |  |  | 0.385 |
| Married | 1891(60.00) | 603(57.85) | 659(62.07) | 629(58.76) |  |
| Others | 897(22.77) | 315(24.86) | 283(20.95) | 299(23.75) |  |
| Never married | 566(17.22) | 200(17.29) | 176(16.99) | 190(17.50) |  |
| **Smoking status, n (%)** |  |  |  |  | 0.108 |
| Never | 1790(51.99) | 571(51.25) | 580(49.39) | 639(55.97) |  |
| Current | 789(25.21) | 285(27.86) | 266(25.75) | 238(22.64) |  |
| Former | 775(22.80) | 262(20.89) | 272(24.86) | 241(21.39) |  |
| BMI (kg/m^2^) | 27.32(24.16,31.13) | 24.64(22.25,27.47) | 26.45(23.88,29.74) ^*^ | 31.05(27.64,36.55) ^#£^ | <0.001 |
| WC (cm) | 95.81(86.60,105.90) | 88.58(80.90,96.20) | 93.50(84.80,101.50) ^*^ | 106.41(96.44,117.42) ^#£^ | <0.001 |
| SBP (mmHg) | 119.33(110.00,130.00) | 116.00(107.33,126.67) | 118.14(108.67,130.67) ^*^ | 122.00(114.00,132.00) ^#£^ | <0.001 |
| DBP (mmHg) | 72.63±10.83 | 69.12±10.81 | 72.79±10.08^*^ | 74.87±11.18^#£^ | <0.001 |
| Hypertension, n (%) | 976(25.20) | 277(20.60) | 273(20.82) | 426(34.26) ^#£^ | <0.001 |
| Diabetes, n (%) | 364(7.98) | 59(3.17) | 71(4.02) | 234(16.64) ^#£^ | <0.001 |
| Antihypertensive drugs, n (%) | 652(17.06) | 176(12.36) | 179(13.64) | 297(24.98) ^#^ | <0.001 |
| Glucose-lowering drugs, n (%) | 187(3.82) | 21(0.94) | 39(1.91) | 127(8.40) ^#£^ | <0.001 |
| Lipid-lowering drugs, n (%) | 363(11.35) | 115(8.72) | 98(9.68) | 150(15.63) ^#£^ | <0.001 |
| HbA1C (%) | 5.30(5.10,5.50) | 5.20(5.00,5.40) | 5.20(5.00,5.40) | 5.50(5.20,5.80) ^#£^ | <0.001 |
| Glucose (mmol/L) | 5.34(4.97,5.77) | 5.15(4.81,5.50) | 5.29(4.95,5.63) ^*^ | 5.63(5.21,6.17) ^#£^ | <0.001 |
| Insulin(pmol/L) | 60.37(43.98,86.94) | 27.12(20.06,34.56) | 56.62(49.02,65.64) ^*^ | 108.70(87.51,145.50) ^#£^ | <0.001 |
| TC (mmol/L) | 5.16±1.10 | 5.12±1.10 | 5.11±1.02 | 5.27±1.18^#£^ | 0.033 |
| LDL-C (mmol/L) | 3.19±0.98 | 2.95±0.95 | 3.24±0.92^*^ | 3.28±1.05^#^ | <0.001 |
| HDL-C (mmol/L) | 1.22(1.03,1.53) | 1.47(1.22,1.81) | 1.23(1.06,1.53) ^*^ | 1.09(0.93,1.27) ^#£^ | <0.001 |
| TG (mmol/L) | 1.30(0.87,1.91) | 0.91(0.67,1.32) | 1.24(0.87,1.68) ^*^ | 1.73(1.23,2.62) ^#£^ | <0.001 |
| eGFR(ml/min/1.73m^2^) | 103.93±31.14 | 92.94±20.74 | 108.84±31.67^*^ | 105.08±34.45^#£^ | <0.001 |

HOMA2-IR homeostatic model assessment, BMI body mass index, WC waist circumference, SBP systolic blood pressure, DBP diastolic blood pressure, HbA1C glycosylated hemoglobin, TC total cholesterol, LDL-C low-density lipoprotein, LDL-C high-density lipoprotein, TG triglycerides, eGFR estimated glomerular filtration rate. Weighted means and standard deviation (SD) for continuous variables. Unweighted numbers and weighted proportions for categorical variables. *: *P* value between tertile1 and tertile2 < 0.05. #: *P* value between tertile1 and tertile3 < 0.05. £: *P* value between tertile2 and tertile3 < 0.05.

**Table S8** The associations of HOMA2-IR with all-cause mortality among participants of propensity score matching.

|  | HOMA2-IR | | | | | | | | *P* for trend |
| --- | --- | --- | --- | --- | --- | --- | --- | --- | --- |
|  | Tertile 1 | | Tertile 2 | | Tertile 3 | | Per SD |  |  |
| BMI<25kg/m^2^ | Ref. |  | 1.08(0.72-1.61) | 0.716 | 1.30(0.86-1.96) | 0.211 | 1.22(0.99-1.51) | 0.063 | 0.209 |
| 25≤BMI<30kg/m^2^ | Ref. |  | 1.27(0.83-1.96) | 0.274 | 0.88(0.60-1.30) | 0.533 | 1.24(1.02-1.52) | 0.035 | 0.347 |
| BMI≥30kg/m^2^ | Ref. |  | 0.58(0.42-0.79) | <0.001 | 0.67(0.41-1.08) | 0.097 | 0.99(0.76-1.30) | 0.967 | 0.107 |

Statistical model was adjusted for age, gender, race, smoking status, education level, family income to poverty ratio, marital status, hypertension, diabetes, BMI, SBP, LDL-C, HDL-C, TG, HbA1c, eGFR.

**Table S9** The associations of HOMA2-IR with CV mortality among participants of propensity score matching.

|  | HOMA2-IR | | | | | | | | *P* for trend |
| --- | --- | --- | --- | --- | --- | --- | --- | --- | --- |
|  | Tertile 1 | | Tertile 2 | | Tertile 3 | | Per SD |  |  |
| BMI<25kg/m^2^ | Ref. |  | 1.50(0.66-3.40) | 0.333 | 1.08(0.50-2.36) | 0.837 | 1.00(0.66-1.53) | 0.996 | 0.996 |
| 25≤BMI<30kg/m^2^ | Ref. |  | 2.20(1.16-4.19) | 0.016 | 1.03(0.49-2.17) | 0.932 | 1.06(0.75-1.49) | 0.748 | 0.819 |
| BMI≥30kg/m^2^ | Ref. |  | 0.99(0.36-2.69) | 0.984 | 1.13(0.33-3.81) | 0.846 | 0.90(0.63-1.28) | 0.546 | 0.850 |

Statistical model was adjusted for age, gender, race, smoking status, education level, family income to poverty ratio, marital status, hypertension, diabetes, BMI, SBP, LDL-C, HDL-C, TG, HbA1c, eGFR.

**Figure S1** The stratified analysis of association between HOMA2-IR and (A) all-cause and (B) CV mortality. Adjusted model included age, gender, race, smoking status, education level, family income to poverty ratio, marital status, hypertension, diabetes, BMI, SBP, LDL-C, HDL-C, TG, HbA1c, eGFR.
